# Supplementary material for: Microbial communities in sediment from Zostera marina patches, but not the Z. marina leaf or root microbiomes, vary in relation to distance from patch edge
Source: PeerJ. 2017 Apr 27;5:e3246. doi: 10.7717/peerj.3246 (PMC5410140; doi:10.7717/peerj.3246)
Supplement: Table S1 — Comparing intra-sample diversity between different sample types (leaf, root, sediment). Kruskal–Wallis tests found differences between intra-sample diversity between different sample types for all metrics (p < 0.001). [file peerj-05-3246-s001.docx]

**Diversity Metric Pairwise Comparison p.adj**

Chao1 leaf-root 1.000000

leaf-sediment 1.554775e-05

root-sediment 3.940622e-04

Observed OTUs leaf-root 1.00000000

leaf-sediment 3.661058e-05

root-sediment 1.842943e-04

Shannon leaf-root 1.0000000

leaf-sediment 1.317658e-04

root-sediment 5.232625e-05

Simpson leaf-root 1.0000000

leaf-sediment 3.181862e-04

root-sediment 1.992357e-05
