# Supplementary material for: Microbial communities in sediment from Zostera marina patches, but not the Z. marina leaf or root microbiomes, vary in relation to distance from patch edge
Source: PeerJ. 2017 Apr 27;5:e3246. doi: 10.7717/peerj.3246 (PMC5410140; doi:10.7717/peerj.3246)
Supplement: Table S2 — Comparing sediment intra-sample diversity between different locations (inside, edge, outside). Kruskal–Wallis tests found differences between intra-sample diversity at different locations for Chao1 and observed OTUs (p < 0.001), but not for Shannon or Simpson indices (p > 0.05). [file peerj-05-3246-s002.docx]

**Diversity Metric Pairwise Comparison p.adj**

Chao1 edge-inside 0.6879958

outside-edge 0.3116285

outside-inside 0.0140332

Observed OTUs edge-inside 1.00000000

outside-edge 0.02162871

outside-inside 0.01403320

Shannon edge-inside 1.0000000

outside-edge 1.0000000

outside -inside 0.8665331

Simpson edge-inside 0.3593848

outside-edge 0.9665964

outside-inside 1.0000000
