# Supplementary material for: Microbial communities in sediment from Zostera marina patches, but not the Z. marina leaf or root microbiomes, vary in relation to distance from patch edge
Source: PeerJ. 2017 Apr 27;5:e3246. doi: 10.7717/peerj.3246 (PMC5410140; doi:10.7717/peerj.3246)
Supplement: Table S3 — Confusion matrix results for random forest classifier using leave-one-out cross validation with 1,000 trees to classify samples by tissue type (leaf, root, sediment). The estimated error of the classifier was 0.05 and the ratio of the baseline error to the observed error was 8.0. [file peerj-05-3246-s003.docx]

**True leaf root sediment class error**

**leaf** 8 0 0 0

**root** 2 6 0 0.25

**sediment** 0 0 24 0
