# Supplementary material for: Microbial communities in sediment from Zostera marina patches, but not the Z. marina leaf or root microbiomes, vary in relation to distance from patch edge
Source: PeerJ. 2017 Apr 27;5:e3246. doi: 10.7717/peerj.3246 (PMC5410140; doi:10.7717/peerj.3246)
Supplement: Table S4 — Confusion matrix results for random forest classifier using leave-one-out cross validation with 1,000 trees to classify sediment samples by location (inside, edge, outside). The estimated error of the classifier was 0.125 and the ratio of the baseline error to the observed error was 5.33. [file peerj-05-3246-s004.docx]

**True inside edge outside class error**

**inside** 6 2 0 0.25

**edge** 1 7 0 0.125

**outside** 0 0 8 0
