# Supplementary material for: Microbial communities in sediment from Zostera marina patches, but not the Z. marina leaf or root microbiomes, vary in relation to distance from patch edge
Source: PeerJ. 2017 Apr 27;5:e3246. doi: 10.7717/peerj.3246 (PMC5410140; doi:10.7717/peerj.3246)
Supplement: Table S5 — Mean and standard deviation of the relative abundance of each taxonomic class of bacteria for leaves, roots and rhizosphere sediment. Only classes that have a mean relative abundance of at least one percent are included here. [file peerj-05-3246-s005.docx]

| **Sample Type** | **Phylum** | **Class** | **Mean** | **Standard Deviation** |
| --- | --- | --- | --- | --- |
| **Leaf** | Proteobacteria | Gammaproteobacteria | 20.54089106 | 7.303316493 |
|  | Firmicutes | Clostridia | 16.49755874 | 12.00207282 |
|  | Bacteroidetes | Bacteroidia | 12.606805 | 8.579568001 |
|  | Proteobacteria | Alphaproteobacteria | 11.40906317 | 8.530960351 |
|  | Bacteroidetes | Flavobacteriia | 7.815837656 | 4.737802641 |
|  | Bacteroidetes | Saprospirae | 7.712847116 | 4.206482962 |
|  | Firmicutes | Bacilli | 3.112602991 | 2.216421897 |
|  | Chloroflexi | Anaerolineae | 2.811260299 | 2.358183598 |
|  | Proteobacteria | Betaproteobacteria | 2.635794934 | 1.126538091 |
|  | Proteobacteria | Deltaproteobacteria | 2.567134574 | 1.572368594 |
|  | Planctomycetes | Planctomycetia | 2.56332011 | 2.363962273 |
|  | Proteobacteria | Epsilonproteobacteria | 0.62557217 | 0.311200459 |
|  | WS3 | PRR-12 | 0.064845896 | 0.050109263 |
|  | Spirochaetes | Spirochaetes | 0.026701251 | 0.038035504 |
| **Root** | Proteobacteria | Epsilonproteobacteria | 17.93179738 | 15.551616 |
|  | Proteobacteria | Deltaproteobacteria | 13.40784254 | 11.29031917 |
|  | Bacteroidetes | Bacteroidia | 12.79752823 | 8.798177101 |
|  | Proteobacteria | Gammaproteobacteria | 12.59536161 | 11.01258923 |
|  | Firmicutes | Clostridia | 8.002746414 | 8.373258004 |
|  | Bacteroidetes | Flavobacteriia | 6.118400977 | 6.105148108 |
|  | Proteobacteria | Alphaproteobacteria | 4.836740922 | 6.285771829 |
|  | Chloroflexi | Anaerolineae | 3.730546231 | 2.328739584 |
|  | Planctomycetes | Planctomycetia | 2.418370461 | 4.298578675 |
|  | Proteobacteria | Betaproteobacteria | 1.907232225 | 2.8665752 |
|  | Bacteroidetes | Saprospirae | 1.335062557 | 1.356270677 |
|  | Spirochaetes | Spirochaetes | 1.289288984 | 1.299364555 |
|  | Firmicutes | Bacilli | 1.14052487 | 1.101581407 |
|  | WS3 | PRR-12 | 0.663716814 | 0.80485329 |
| **Rhizosphere Sediment** | Proteobacteria | Gammaproteobacteria | 18.16829417 | 3.351858851 |
|  | Proteobacteria | Deltaproteobacteria | 14.90120537 | 2.574936231 |
|  | Bacteroidetes | Bacteroidia | 13.26670735 | 2.306000298 |
|  | Bacteroidetes | Flavobacteriia | 9.335901739 | 3.364477583 |
|  | Firmicutes | Clostridia | 5.111382362 | 3.254106782 |
|  | Chloroflexi | Anaerolineae | 3.871681416 | 1.635250425 |
|  | Proteobacteria | Epsilonproteobacteria | 3.076365578 | 1.138483986 |
|  | WS3 | PRR-12 | 2.252441257 | 0.407773208 |
|  | Proteobacteria | Alphaproteobacteria | 1.916768386 | 0.63476784 |
|  | Spirochaetes | Spirochaetes | 1.733674092 | 0.474218377 |
|  | Bacteroidetes | Saprospirae | 1.4914556 | 0.919792805 |
|  | Planctomycetes | Planctomycetia | 1.169133354 | 0.261966058 |
|  | Firmicutes | Bacilli | 0.871605127 | 0.701902919 |
|  | Proteobacteria | Betaproteobacteria | 0.078196521 | 0.064943054 |
