# Supplementary material for: Microbial communities in sediment from Zostera marina patches, but not the Z. marina leaf or root microbiomes, vary in relation to distance from patch edge
Source: PeerJ. 2017 Apr 27;5:e3246. doi: 10.7717/peerj.3246 (PMC5410140; doi:10.7717/peerj.3246)
Supplement: Table S6 — Comparing environmental factors between different locations (inside, edge, outside). [file peerj-05-3246-s006.docx]

**Factor F value Pr(>F)**

Carbon:Nitrogen Ratio 5.013 0.0344

Total Inorganic Carbon (%) 4.714 0.0398

Total Organic Carbon (%) 4.183 0.0519

Eelgrass Density 491.7 6.44e-10

Dissolved Oxygen (%) 40.62 3.12e-05

pH 4.833 0.0375

Salinity (per mil) 0.808 0.476

Temperature (°C) 0.346 0.716
