# Supplementary material for: Microbial communities in sediment from Zostera marina patches, but not the Z. marina leaf or root microbiomes, vary in relation to distance from patch edge
Source: PeerJ. 2017 Apr 27;5:e3246. doi: 10.7717/peerj.3246 (PMC5410140; doi:10.7717/peerj.3246)
Supplement: Table S7 — Comparing sediment size fractions between different locations (inside, edge, outside). [file peerj-05-3246-s007.docx]

**Sediment Size Fraction F value Pr(>F)**

710 μm 6.559 0.0175

500 μm 2.757 0.116

355 μm 1.149 0.359

250 μm 1.46 0.282

180 μm 2.463 0.14

90 μm 1.152 0.359

63 μm 10.62 0.00428

Silt 2.786 0.114
