# Supplementary material for: Microbial communities in sediment from Zostera marina patches, but not the Z. marina leaf or root microbiomes, vary in relation to distance from patch edge
Source: PeerJ. 2017 Apr 27;5:e3246. doi: 10.7717/peerj.3246 (PMC5410140; doi:10.7717/peerj.3246)
Supplement: Table S8 — Comparing environmental factors (p < 0.055) between different locations (inside, edge, outside). [file peerj-05-3246-s008.docx]

**Factor Pairwise Location p adj**

Carbon:Nitrogen Ratio inside-edge 0.5951735

outside-edge 0.1443355

outside-inside 0.0308486

Total Inorganic Carbon (%) inside-edge 0.1021810

outside-edge 0.0424658

outside-inside 0.8409400

Total Organic Carbon (%) inside-edge 0.1960852

outside-edge 0.0463592

outside-inside 0.6257670

Eelgrass Density inside-edge 0.0000001

outside-edge 0.0000008

outside-inside 0

Dissolved Oxygen (%) inside-edge 0.0000263

outside-edge 0.0005767

outside-inside 0.0415206

pH inside-edge 0.0486224

outside-edge 0.0720466

outside-inside 0.9655583
