# Supplementary material for: Microbial communities in sediment from Zostera marina patches, but not the Z. marina leaf or root microbiomes, vary in relation to distance from patch edge
Source: PeerJ. 2017 Apr 27;5:e3246. doi: 10.7717/peerj.3246 (PMC5410140; doi:10.7717/peerj.3246)
Supplement: Table S9 — Comparing sediment size fractions (p < 0.05) between different locations (inside, edge, outside). [file peerj-05-3246-s009.docx]

**Sediment Size Fraction Pairwise Location p adj**710 μm inside-edge 0.6075766
 outside-edge 0.0164467
 outside-inside 0.0750780
63 μm inside-edge 0.0984172
 outside-edge 0.1149065
 outside-inside 0.0032738
