# Supplementary figures and images for: Microbial communities in sediment from Zostera marina patches, but not the Z. marina leaf or root microbiomes, vary in relation to distance from patch edge
Source: PeerJ. 2017 Apr 27;5:e3246. doi: 10.7717/peerj.3246 (PMC5410140; doi:10.7717/peerj.3246)

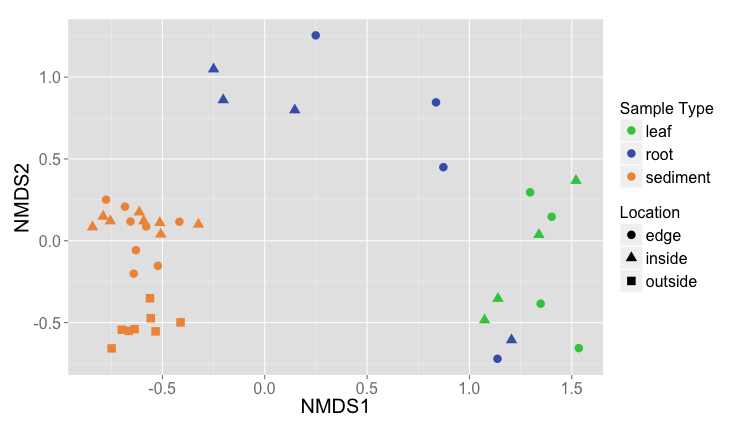

Supplement: Figure S1 — Bray Curtis dissimilarities of microbial communities found in samples are shown here colored by sample type (leaf, root, sediment) with different shapes for location (inside, edge, outside). [file peerj-05-3246-s010.png]

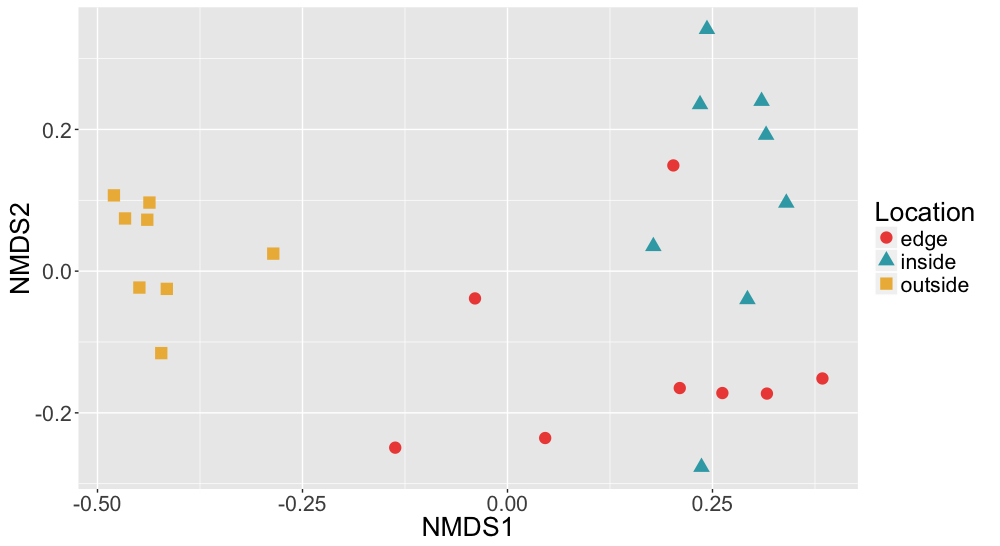

Supplement: Figure S2 — Bray Curtis dissimilarities of microbial communities found in sediment samples are shown here colored by location (inside, edge, outside). [file peerj-05-3246-s011.png]

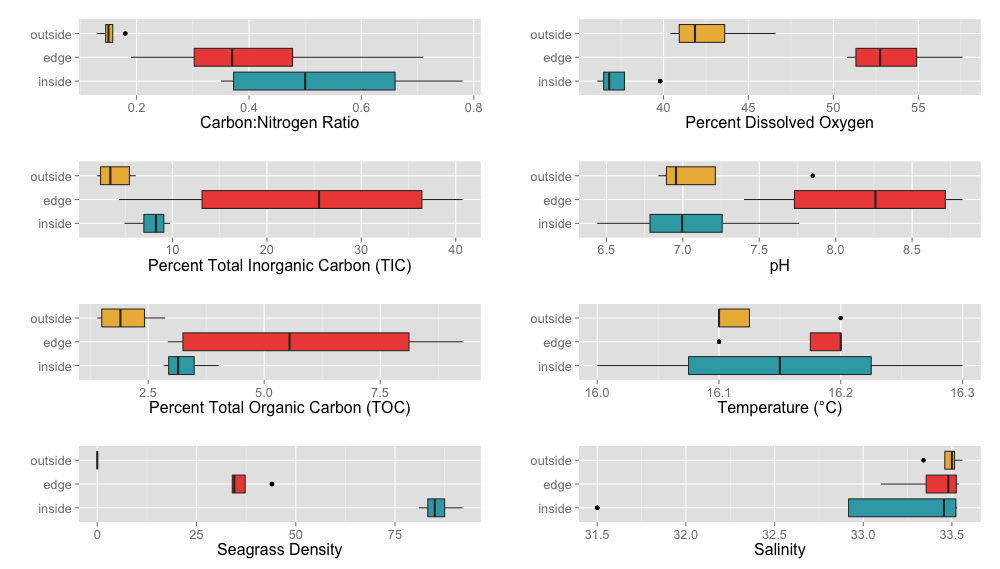

Supplement: Figure S3 — A variety of environmental factors were measured during the course of this project including Carbon:Nitrogen ratio, percent total inorganic Carbon (TIC), percent total organic Carbon (TOC), eelgrass density (number of shoots), percent dissolved oxygen, pH, temperature (°C) and salinity (per mil). These measurements are shown above split between different locations (inside, edge, outside) as boxplots. [file peerj-05-3246-s012.png]

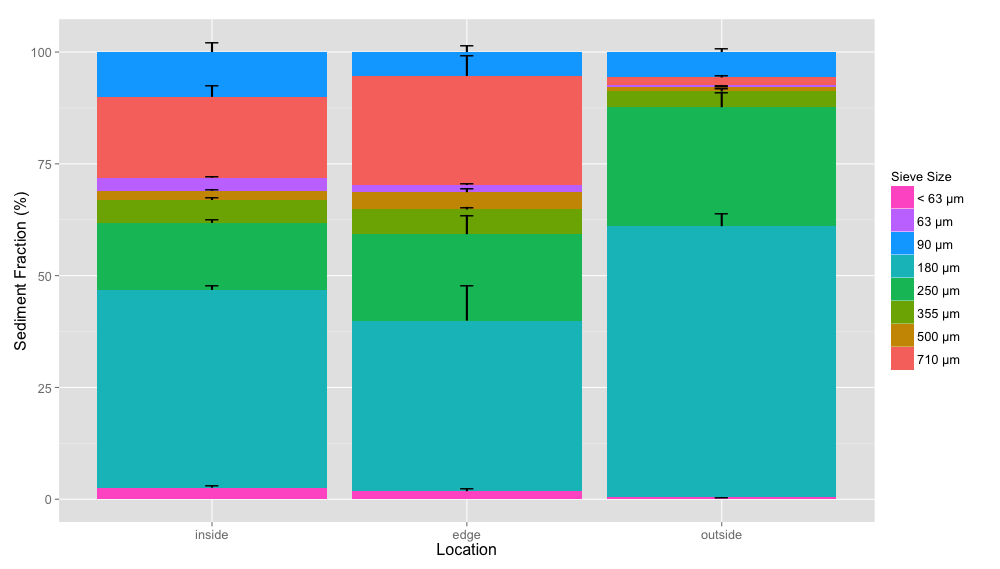

Supplement: Figure S4 — Average sediment size composition for each location (inside, edge, outside) colored by sieve size fractions with standard error bars. [file peerj-05-3246-s013.png]
